# Leukotriene B4 receptor knockdown affects PI3K/AKT/mTOR signaling and apoptotic responses in colorectal cancer

**DOI:** 10.17305/bb.2024.10119

**Published:** 2024-08-01

**Authors:** Cui Tang, Aili Wang, YanLin Zhao, WenYing Mou, Jun Jiang, Jie Kuang, Bin Sun, Erjiang Tang

**Affiliations:** 1 Department of Radiology, Yangpu Hospital, School of Medicine, Tongji University, Shanghai, China; 2 Center for Clinical Research and Translational Medicine, Yangpu Hospital, School of Medicine, Tongji University, Shanghai, China; 3 Endoscopy Center, Minhang District Central Hospital of Fudan University, Shanghai, China

**Keywords:** Leukotriene B4 receptor (LTB4R), colorectal cancer (CRC), phosphoinositide 3-kinase/protein kinase B/mammalian target of rapamycin (PI3K/AKT/mTOR) signaling pathway, apoptosis.

## Abstract

Colorectal cancer (CRC) presents a landscape of intricate molecular dynamics. In this study, we focused on the role of the leukotriene B4 receptor (LTB4R) in CRC, exploring its significance in the disease’s progression and potential therapeutic approaches. Using bioinformatics analysis of the GSE164191 and the Cancer Genome Atlas-colorectal adenocarcinoma (TCGA-COAD) datasets, we identified *LTB4R* as a hub gene influencing CRC prognosis. Subsequently, we examined the relationship between LTB4R expression, apoptosis, and the phosphoinositide 3-kinase/protein kinase B/mammalian target of rapamycin (PI3K/AKT/mTOR) signaling pathway through cellular and mice experiments. Our findings revealed that LTB4R is highly expressed in CRC samples and is pivotal for determining prognosis. In vitro experiments demonstrated that silencing LTB4R significantly impeded CRC cell viability, migration, invasion, and colony formation. Correspondingly, in vivo tests indicated that LTB4R knockdown led to markedly slower tumor growth in mice models. Further in-depth investigation revealed that LTB4R knockdown significantly amplified the apoptosis in CRC cells and upregulated the expression of apoptosis-related proteins, such as caspase-3 and caspase-9 while diminishing p53 expression. Interestingly, silencing LTB4R also resulted in a significant downregulation of the PI3K/AKT/mTOR signaling pathway. Moreover, pretreatment with the PI3K activator 740Y-P only partially attenuated the effects of LTB4R knockdown on CRC cell behavior, emphasizing LTB4R’s dominant influence in CRC cell dynamics and signaling pathways. LTB4R stands out as a critical factor in CRC progression, profoundly affecting cellular behavior, apoptotic responses, and the PI3K/AKT/mTOR signaling pathway. These findings not only shed light on LTB4R’s role in CRC but also establish it as a potential diagnostic biomarker and a promising target for therapeutic intervention.

## Introduction

Colorectal cancer (CRC), originating from the colon or rectum, is characterized by uncontrolled cell growth and tissue invasion [1, 2]. Globally, the diagnosis and mortality rates of CRC have alarmingly increased, impacting both developed and developing nations [3]. As of 2020, CRC accounts for 10% of all cancer cases worldwide, and its mortality rate stands at 9.4% of all cancer deaths, ranking just below lung cancer at 18%. Projections based on aging trends, population growth, and human social development patterns suggest that by 2040, there will be 3.2 million new CRC cases worldwide. The rise in CRC incidence stems mainly from lifestyle and dietary shifts toward westernization, increasing exposure to environmental risk factors [4, 5]. The complex etiology of CRC intertwines genetic predispositions, lifestyle factors, and environmental exposures [6]. Recognized modifiable risk factors, such as obesity, smoking, and diabetes, show similar trends in the prevalence as seen in early-onset CRC. Associated non-modifiable risk factors include sex, race, inflammatory bowel disease (IBD), and family history of CRC [7]. These multifaceted interactions culminate in the onset and progression of the disease. Existing treatment options for CRC encompass surgical resection and systemic interventions such as chemotherapy, radiotherapy, and molecularly targeted therapy in advanced stages [8, 9]. However, the heterogeneity of CRC makes certain standard treatments suboptimal for specific patient subgroups [10]. This variability in results underscores the importance of early detection and a personalized medicine approach. In light of this context, the pursuit of novel diagnostic biomarkers, enhanced treatment modalities, and reliable prognostic indicators has become paramount. These efforts are geared toward early detection, precise interventions, and the enhancement of patient trajectories.

The leukotriene B4 receptor (LTB4R), a G protein-coupled receptor, specifically interacts with leukotriene B4, orchestrating a multitude of cellular processes [11]. Its pivotal role in the pathogenesis and progression of cancer has swiftly propelled it to prominence within oncology research [12, 13]. Recent studies underscore the centrality of LTB4R in cancer research, owing to its multifarious roles. For instance, Long et al. [14] identified both prognostic and immunological significance of LTB4R across diverse cancer types. Furthermore, research by Ma et al. [15] pinpoints its association with CRC prognosis. The importance of this receptor in CRC continues to be solidified through insights into its impact on inflammation-induced tumorigenesis. Moreover, LTB4R’s involvement in signaling cascades, particularly the phosphoinositide 3-kinase/protein kinase B/mammalian target of rapamycin (PI3K/AKT/mTOR) signaling pathway, enhanced its role in regulating CRC cell viability and proliferation. Numerous mutations linked to cancer development, affecting various pathways, have been identified to date. Among them, the PI3K/AKT/mTOR signaling pathway exhibits aberrant expression in a variety of contexts. This pathway is believed to play a key role in cancer cell proliferation, migration, invasion, and, more recently, in the development of chemoresistance [16]. This signaling pathway is one of the most extensively studied intracellular pathways, with its dysregulation being validated in a variety of tumors, including ovarian cancer [17]. The PI3K/AKT/mTOR signaling pathway is also integral in the study of small molecule compounds. In a recent study, rutin, a flavonoid found in fruits and vegetables, demonstrated antitumor effects both in vivo and in vitro at different doses by modulating the mTOR signaling pathway [18]. In CRC, frequent mutations in both the PI3K pathway and the mitogen-activated protein kinase (MAPK) pathway promote tumor progression often alongside other common mutations in the wingless-type MMTV integration site family (WNT) signaling pathway, p53 and transforming growth factor beta (TGFβ) signaling pathways [19]. Notably, among non-hypermutated tumors, which represent 90% of all CRCs, approximately 59% of metastatic CRC patients exhibit mutations in the MAPK pathway, and 26.7% of these patients show concurrent mutations in the PI3K/AKT/mTOR pathway [20]. Unraveling the complex interplay between LTB4R and various cellular processes could lead to the reveal of new therapeutic targets and prognostic markers.

Based on bioinformatics analysis and differential gene assessment, we delved into the complex molecular landscape supporting CRC progression. Recognizing the importance of LTB4R in CRC prognosis, our objective was to elucidate the multiple roles and regulatory dynamics associated with its gene. We concentrated on the molecular interactions and cellular consequences of LTB4R regulation, with particular emphasis on its relationships with the PI3K signaling cascade and the apoptotic pathway. The aim of this study was not only to elucidate these intricate relationships but also to pave the way for new therapeutic avenues, thereby enriching the current understanding of the complex molecular structure of CRC.

## Materials and methods

### Analysis of differentially expressed genes (DEGs) in public databases

The GSE164191 dataset, sourced from the Gene Expression Omnibus (GEO) database (https://www.ncbi.nlm.nih.gov/gds/), encompassed 59 samples from the case group and 62 samples from the control group. Additionally, we extracted 455 colorectal adenocarcinoma (COAD) samples and 41 control samples from The Cancer Genome Atlas (TCGA) database (https://tcga-data.nci.nih.gov/tcga). To identify DEGs in both datasets, specific algorithms tailored to each data source were employed. For the GSE164191 dataset, the GEO2R tool was utilized. Meanwhile, for the TCGA dataset, the “limma” package in the R language was adapted to identify DEGs. In both instances, the DEGs were selected based on a consistent criterion: a fold change (FC) of either > 1.3 or < 0.77, with a significance threshold set at *P* < 0.05. Following identification, the DEGs from the GSE164191 dataset were visualized using the ggplot2 package in R, resulting in detailed volcano plots that effectively illustrate the distribution and significance of these DEGs.

### Weighted Gene Co-Expression Network Analysis (WGCNA)

Following the identification of DEGs from the GSE164191 dataset, we proceeded with a detailed network analysis. To this end, a gene co-expression network tailored specific to these DEGs was constructed utilizing the WGCNA package in R. The process began with the computation of a correlation matrix for all gene pairs, capturing their mutual relationships within the identified DEGs. To ensure a scale-free network topology, an appropriate soft-thresholding power was determined. With the optimized data, hierarchical clustering was applied to group genes into modules based on their patterns of co-expression. Subsequently, the signature genes of each module were correlated with clinical characteristics (specifically, GSE164191-case or GSE164191-control status) to discern modules of clinical relevance. Finally, visualization tools within the WGCNA package were employed to graphically represent the overall network, highlight module-trait associations, and detail connections within individual modules.

### Functional enrichment analysis of overlapping genes between TCGA upregulated DEGs and the turquoise module

To identify genes common to both TCGA-DEGs and the turquoise modules, we performed overlap analysis using the “VennDiagram” package in R. Given that genes within the turquoise module were upregulated, we paid particular attention on the genes that were upregulated in the TCGA dataset. Following this overlap identification, we sought to elucidate the underlying molecular mechanisms underpinning these overlapping genes. To facilitate this, we utilized the Database for Annotation, Visualization and Integrated Discovery (DAVID) (https://david.ncifcrf.gov/) for conducting WikiPathway (WP) and Gene Ontology (GO) enrichment analyses. The GO analysis encompassed three main categories: biological process (BP), cellular component (CC), and molecular function (MF). The threshold for statistically significant outcomes in the enrichment analyses was set at *P* < 0.05.

### Establishment of a prognostic risk model

We employed the least absolute shrinkage and selection operator (LASSO) regression analysis, a powerful computational method commonly used in bioinformatics research. This analysis was performed on a subset of 103 overlapping genes to determine the optimal minimum λ value. The expression levels of these selected overlapping genes were used to compute individual risk scores. Subsequently, we generated survival scatterplots and expression heatmaps to visualize the performance of the prognostic risk model. This analysis revealed that 11 genes were significantly associated with the established risk models. We then separated the TCGA samples into high-risk and low-risk groups based on their mean risk scores, as a means to verify the prognostic risk model. To assess the prognostic value of our model further, we conducted an overall survival (OS) analysis using Kaplan–Meier (KM) methods. Additionally, receiver operating characteristic (ROC) analysis was performed to evaluate the model’s predictive performance. By comparing the area under the curve (AUC) values from these analyses, we identified the most effective prognostic model, enhancing our ability to predict patient outcomes.

### Prognostic assessment of the 11 significant genes

Following the identification of 11 significant genes from the prognostic risk model, we examined their expression patterns within the GSE164191 dataset and TCGA-COAD samples. Subsequently, the prognostic significance of each gene was assessed by generating KM survival curves. The log-rank test was used to investigate the relationship between gene expression levels and OS rate among patients. This test’s *P* values were particularly instrumental in discerning differential survival outcomes between the defined groups, based on their gene expression levels. Genes that demonstrated a statistically significant *P* value of less than 0.05 in this analysis were considered to potentially influence patient survival outcomes.

### Cell culture

In our research, we utilized four distinct cell lines: SW480, HCT-116, and RKO, which are human CRC cell lines, and CDC-18Co, a normal human colon fibroblast cell line. These cell lines were obtained from the American Type Culture Collection (ATCC). We cultured them in Dulbecco’s Modified Eagle Medium (DMEM) (Gibco) supplemented with 10% fetal bovine serum (FBS) (Thermo Fisher Scientific) and 1% penicillin–streptomycin (Gibco). The maintenance of these cells was carried out at a constant temperature of 37 ^∘^C in a humidified atmosphere containing 5% CO_2_.

### Cell transfection and treatment

For transient transfection, CRC cells were seeded in 24-well plates at a density of 2×10^4^ cells per well. The cells were transfected with two distinct small interfering RNAs (siRNAs), designated si-LTB4R-1 and si-LTB4R-2, aimed at reducing the LTB4R expression. A non-targeting siRNA (si-negative control [NC]) was used as the control. The transfection was conducted using Lipofectamine 3000 (Invitrogen, USA), following the manufacturer’s instructions. After the transfection process, the cells were incubated for a specific period to ensure effective knockdown of LTB4R expression. Additionally, the CRC cells were treated with 740Y-P, a phosphopeptide activator frequently used in research for its role in modulating cellular pathways [21, 22]. This treatment was conducted to assess potential synergistic or antagonistic effects that might arise from the combination of LTB4R knockdown and the activation of cellular pathways by 740Y-P.

### Mouse model

We employed HCT-116 and RKO cells, along with five-week-old nude mice, to establish a subcutaneous xenograft model. Both cell lines, HCT-116 and RKO, previously transfected with either si-NC or si-LTB4R-1, were cultured in vitro. These cells were suspended in a 1:1 mixture of Matrigel and Roswell Park Memorial Institute 1640 (RPMI1640) medium. This suspension was then subcutaneously injected into the mice’s abdominal region, with each mouse receiving approximately 1×10^7^ cells. Post-injection, the growth of the tumors derived from HCT-116 and RKO cells was closely monitored. Photographs of the tumors were periodically captured and their volumes were meticulously measured to assess the progression.

### Quantitative real-time polymerase chain reaction (qRT-PCR) assay

Total RNA was extracted from both the experimentally treated cellular samples and the harvested mouse tumor tissues utilizing TRIzol reagent, sourced from Invitrogen. Subsequently, reverse transcription was performed to synthesize complementary DNA (cDNA), utilizing Invitrogen’s SuperScript III First-Strand Synthesis System. The qRT-PCR analyses were conducted on the Applied Biosystems’ StepOnePlus Real-Time PCR System, employing their SYBR Green Master Mix. The expression levels of the target messenger RNA (mRNA) were normalized to glyceraldehyde 3-phosphate dehydrogenase (GAPDH), which served as the control. These levels were calculated using the 2^-ΔΔCt^ method. The specific primers used for the amplification process were as follows: for *LTB4R*, the forward primer was 5′-GACGGTGCATTACCTGTGC-3′, and the reverse primer was 5′-AGTCTTGTCCGCCAAGGTC-3′; for *GAPDH*, the forward primer was 5 5′-TGGTGAAGCAGGCATCTGA-3′, and the reverse primer was 5′-TGCTGTTGAAGTCGCAGGAG-3′.

### Western blotting (WB) assay

For protein sample preparation, we lysed the cells using radioimmunoprecipitation assay (RIPA) buffer from Thermo Fisher Scientific, augmented with protease and phosphatase inhibitors from Roche. The protein concentrations were quantified using the bicinchoninic acid assay (BCA) kit also provided by Thermo Fisher Scientific. Subsequently, equal amounts of each protein sample were separated through the sodium dodecyl sulfate-polyacrylamide gel electrophoresis (SDS-PAGE) and subsequently transferred onto polyvinylidene difluoride (PVDF) membranes from Millipore. The membranes were first blocked, and then probed with primary antibodies against LTB4R, caspase-3, caspase-9, p53, PI3K, phosphorylated-PI3K (p-PI3K), AKT, phosphorylated-AKT (p-AKT), mTOR, and phosphorylated-mTOR (p-mTOR), all at a dilution of 1:2000. GAPDH served as a loading control and was detected with its specific antibody at the same dilution (1:2000) (Sigma-Aldrich). After the incubation with primary antibodies, the membranes were treated with appropriate horseradish peroxidase-conjugated secondary antibodies. The detection of protein bands was achieved using enhanced chemiluminescence (ECL) reagents (Thermo Fisher Scientific).

### Counting Kit-8 (CCK-8) assay

The cells were seeded into 96-well plates at an appropriate density to facilitate proper adhesion. After predetermined time intervals (0, 24, 48, 72, 96, and 120 h), CCK-8 reagent was added to each well. Subsequently, the plates were incubated for an additional 2 h at a temperature of 37^∘^C. Following the incubation period, the absorbance of each well was measured at a wavelength of 450 nm using a BioTek microplate reader.

### Flow cytometry assay

To quantify apoptosis, cells were stained using the Annexin V-fluorescein isothiocyanate (FITC)/propidium iodide (PI) Apoptosis Detection Kit (BD Biosciences), in accordance with the manufacturer’s instructions. Post-staining, cells were instantly examined using a BD FACSCanto II flow cytometer. The ability to determine the percentages of early and late apoptotic cells, as revealed by the Annexin V-FITC and PI staining patterns, facilitated a thorough analysis of the cell death dynamics within the samples.

### Colony formation assay

To evaluate the proliferative potential of RKO and HCT-116 CRC cells, we conducted a colony formation assay. The cells were seeded in 6-well plates, populating each well with 500 cells. Following a 10-day incubation period, the cells were fixed with methanol and then stained using crystal violet. The resulting visible colonies were then counted and examined using an inverted microscope that was equipped with imaging software.

### Transwell assay

For cell migration and invasion assays, Transwell Inserts with Pore Size (Corning) were utilized. HCT-116 and RKO cells were placed in the upper chamber in serum-free DMEM, while the lower chamber contained a medium with 10% FBS for migration assays. For invasion assays, the upper chamber was pre-coated with Matrigel in medium (BD Biosciences). After a 24-h incubation, cells on the membrane’s underside were fixed with 4% paraformaldehyde and stained with 4’,6-diamidino-2-phenylindole (DAPI) to visualize the nuclei. The counting of migrated and invaded cells was performed under a light microscope.

### Ethical statement

This study was reviewed and approved by the Animal Welfare and Ethics Group at the Department of Laboratory Animal Science, Fudan University (Grant No. 2023JS MZX-313).

### Statistical analysis

Statistical analyses were conducted utilizing GraphPad Prism software, version 8.0. Data were presented as mean values accompanied by the standard deviations. The significance level was set at a *P* value of less than 0.05. For statistical testing, one-way analysis of variance (ANOVA), supplemented with subsequent Tukey’s post hoc analysis, was utilized.

## Results

### WGCNA reveals the key module in the GSE164191 dataset

**Figure 1.**
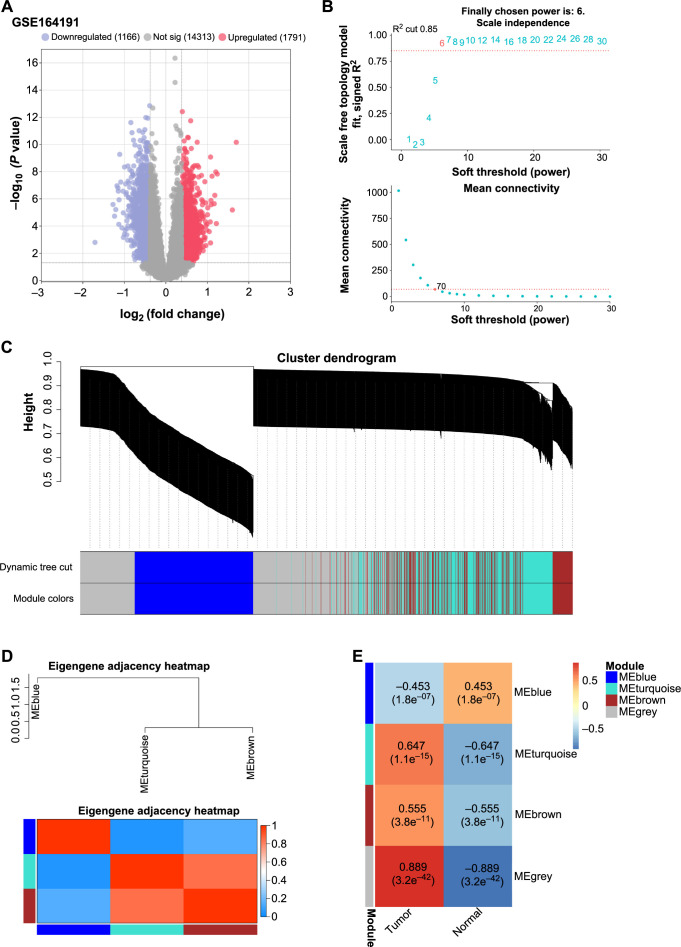
**WGCNA of the GSE164191 dataset identifying key DEGs modules in CRC.** (A) Volcano plot illustrating 1791 upregulated and 1166 downregulated DEGs in GSE164191 samples; (B) Analysis of soft-thresholding power for network topology. The scale-free fit index is plotted on the vertical axis against various soft-thresholding powers on the horizontal axis. The red line demarcates the cutoff for the scale-free topology model fit index at 0.85, achieved at a power of 6; (C) Hierarchical clustering dendrogram of genes, with a color-coded row beneath the dendrogram indicating the assignment of genes to different modules; (D) Eigengene heatmap depicting inter-module adjacency. The intensity of the color represents the strength of correlation between different modules; (E) Heatmap showing the correlation between gene modules and the CRC phenotype. Each cell contains the correlation coefficient and the corresponding *P* value. WGCNA: Weighted Gene Co-expression Network Analysis; DEGs: Differentially expressed genes; CRC: Colorectal cancer; ME: Modul eigengene.

Utilizing the GSE164191 dataset, we embarked on an in-depth exploration of DEG patterns in the context of CRC. As depicted in Figure 1A, 1791 upregulated DEGs along with 1166 downregulated DEGs were identified in CRC and control samples. To discern the most significant DEG clusters, we adopted the WGCNA approach. For the construction of co-expression modules, we set parameters to achieve average connectivity and a scale-free topology model fit index of 0.85 at a soft-thresholding power of 6 (Figure 1B). Each module was assigned a unique color for differentiation (Figure 1C). To ensure the robustness of the modular structure, we merged closely related branches of the dendrogram (Figure 1D). Subsequent analysis revealed a substantial positive correlation between the MEturquoise gene module and CRC (*r* ═ 0.647; Figure 1E). These findings demonstrate the effectiveness of WGCNA in isolating gene modules closely associated with the disease, offering a deeper insight into the genetic underpinnings of CRC.

### Functional enrichment of 103 overlapping genes

**Figure 2.**
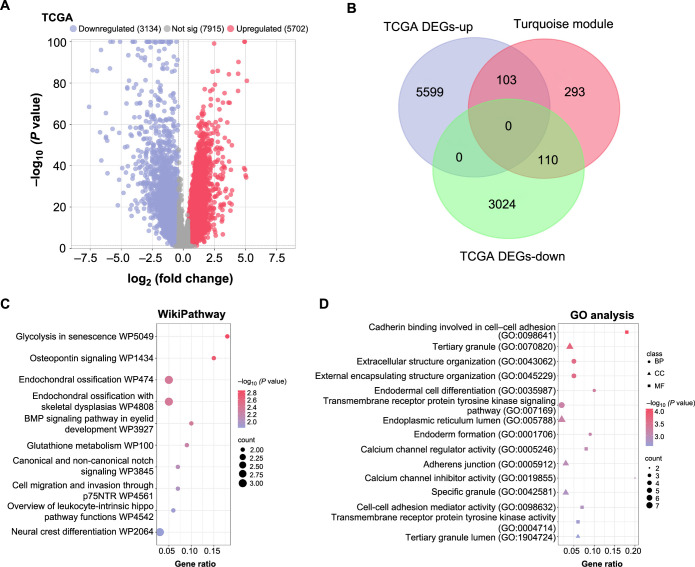
**Functional enrichment analysis of overlapping genes in TCGA-COAD and the turquoise module.** (A) Gene differential analysis in the TCGA-COAD dataset identifying significant expression changes, with 5702 upregulated and 3134 downregulated DEGs observed in CRC compared to control samples; (B) Venn diagram showcasing the intersection of 5702 upregulated DEGs from TCGA-COAD with genes in the turquoise module, resulting in 103 overlapping genes; (C) Bubble plot illustrating the WikiPathway enrichment analysis for the 103 overlapping genes. Each bubble corresponds to a specific pathway, with the size indicating the number of genes involved in that pathway; (D) Bubble plot depicting the GO term enrichment analysis. The plot uses different symbols for each GO category: Dots representing BP, triangles for CC, and squares for MF. TCGA: The Cancer Genome Atlas; COAD: Colorectal adenocarcinoma; CRC: Colorectal cancer; DEGs: Differentially expressed genes; GO: Gene Ontology; BPs: Biological processes; CC: Cellular components; MF: Molecular functions; WP: WikiPathway; BMP: Bone morphogenetic protein; p75NTR: p75 neurotrophin receptor.

We screened DEGs in TCGA-COAD and 41 normal control samples and the results revealed 5702 upregulated DEGs and 3134 downregulated DEGs (Figure 2A). We then focused on the 5702 upregulated DEGs and intersected them with genes from the turquoise module, identifying 103 overlapping genes (Figure 2B). To elucidate the biological implications of these overlapping genes, we undertook a functional enrichment analysis. The WP enrichment analysis revealed significant involvement of these genes in pathways, such as “glycolysis in senescence WP5049,” “osteopontin signaling WP1434,” and “endochondral ossification WP474” (Figure 2C). Additionally, GO term analysis was performed, which shed light on the roles of these genes in several processes. Notably, we observed their involvement in “cadherin binding involved in cell-cell adhesion” (MF), “tertiary granule” (CC), and “extracellular structure organization” (BP), among others (Figure 2D).

### Analysis of the prognostic value of 11 significant genes in colorectal cancer

**Figure 3.**
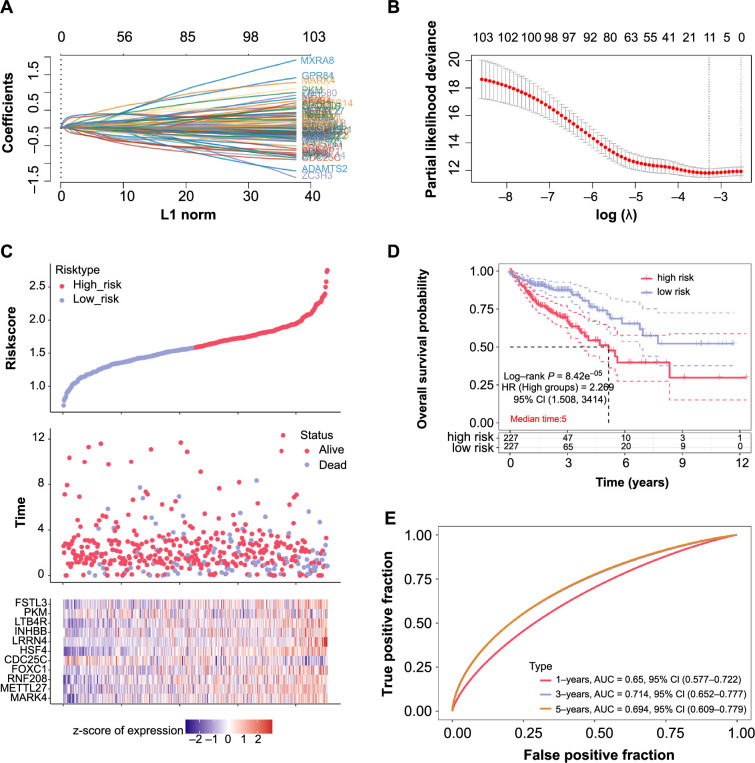
**Prognostic evaluation of 11 significant genes in CRC.** (A) LASSO coefficient profiles for the 103 overlapping genes. The graph plots the L1-norm on the *x*-axis against the corresponding coefficients on the *y*-axis. (B) Selection of the tuning parameter (λ) in the LASSO model using 10-fold cross-validation. The *x*-axis displays Log(*λ*), while the *y*-axis shows the partial likelihood deviance. A vertical line at *λ*_min_ ═ 0.0376 marks the optimal value of *λ*. (C) Risk model visualization. The upper panel depicts the distribution of samples classified as high-risk or low-risk. The middle panel outlines the survival status and duration for each sample. The lower panel presents a clustered heatmap showcasing the expression patterns of the 11 significant genes in both high-risk and low-risk groups. (D) KM survival curves highlighting significant differences in survival rates between high-risk and low-risk groups. The plot provides insights into the median survival over a 5-year period. (E) ROC curves for 1-year, 3-year, and 5-year survival predictions. The AUC values for these periods are 0.65, 0.714, and 0.694, respectively. CRC: Colorectal cancer; LASSO: Least absolute shrinkage and selection operator; KM: Kaplan–Meier; ROC: Receiver operating characteristic; AUC: Area under the curve; FSTL3: Follistatin like 3; PKM: Pyruvate kinase M; LTB4R: Leukotriene B4 receptor; INHBB: Inhibin beta B; LRRN4: Leucine rich repeat neuronal 4; HSF4: Heat shock transcription factor 4; CDC25C: Cell division cycle 25C; FOXC1: Forkhead box C1; RNF208: Ring finger protein 208; METTL27: Methyltransferase like 27; MARK4: Microtubule affinity regulating kinase 4; CI: Confidence interval.

In our efforts to establish a definitive prognostic signature for CRC, we meticulously examined the 103 overlapping genes. Leveraging the LASSO Cox regression method, we identified the most influential genes by selecting the *λ* value that minimized the cross-validation error. This value was determined to be *λ*_min_ ═ 0.0376 (Figure 3A and 3B), leading us to identify 11 genes with significant prognostic value for CRC. Their respective contributions to the risk score model were quantified as follows: Risk score ═ (0.0469) × microtubule affinity regulating kinase 4 (*MARK4*) + (0.0654) × methyltransferase like 27 (*METTL27*) + (0.0305) × ring finger protein 208 (*RNF208*) + (0.0724) × forkhead box C1 (*FOXC1*) + (−0.0581) × cell division cycle 25C (*CDC25C*) + (0.1984) × heat shock transcription factor 4 (*HSF4*) + (0.2587) × leucine-rich repeat neuronal 4 (*LRRN4*) + (0.0064) × inhibin beta B (*INHBB*) + (0.008) × *LTB4R* + (0.004) × pyruvate kinase M (*PKM*) + (0.0562) × follistatin like 3 (*FSTL3*). The differential expression patterns of these 11 genes in CRC samples are depicted in Figure 3C. Further survival analysis confirmed the efficacy of our established risk model. There was a marked difference in survival outcomes between the high-risk and low-risk groups, with the high-risk group exhibiting a significantly poorer prognosis (Figure 3D). Importantly, the ROC curves for our risk model yielded AUC values of 0.65, 0.714, and 0.694 for 1-year, 3-year, and 5-year survival predictions, respectively, underscoring the model’s predictive accuracy (Figure 3E).

**Figure 4.**
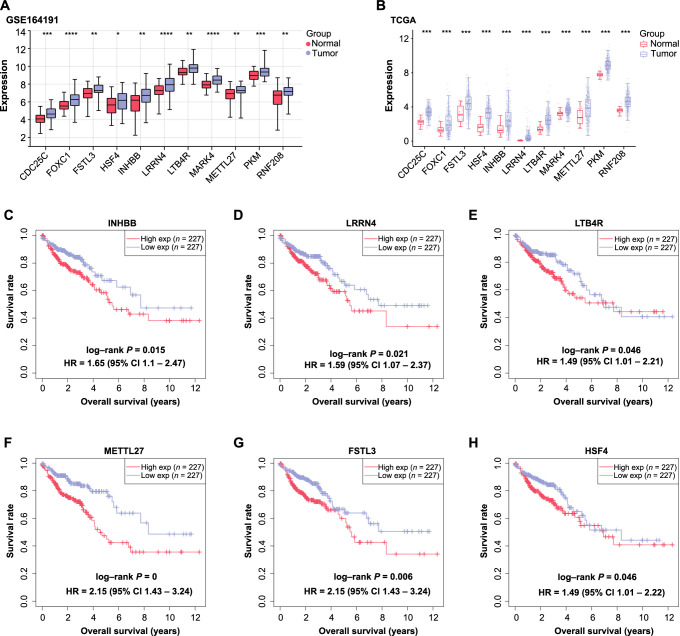
**Clinical relevance and prognostic significance of 11 identified genes in CRC.** (A) Boxplot illustrating the differential expression of 11 prognostic genes in CRC tumor samples compared to normal controls within the GSE164191 dataset. The *y*-axis represents gene expression levels. (B) Boxplot illustrating the differential expression of 11 prognostic genes in CRC tumor samples compared to normal controls within the TCGA dataset. The *y*-axis represents gene expression levels. (C–H) KM survival curves evaluating the prognostic correlations of six significant genes, namely *INHBB* (C), *LRRN4* (D), *LTB4R* (E), *METTL27* (F), *FSTL3* (G), and *HSF4* (H) in CRC patients. The *y*-axis represents OS probability, while the *x*-axis represents time. *P < 0.05; **P < 0.01; ***P < 0.001; ****P < 0.0001. CRC: Colorectal cancer; TCGA: The Cancer Genome Atlas; KM: Kaplan–Meier; INHBB: Inhibin beta B; LRRN4: Leucine rich repeat neuronal 4; LTB4R: Leukotriene B4 receptor; METTL27: Methyltransferase like 27; FSTL3: Follistatin like 3; HSF4: Heat shock transcription factor 4; OS: Overall survival; CDC25C: Cell division cycle 25C; FOXC1: Forkhead box C1; MARK4: Microtubule affinity regulating kinase 4; PKM: Pyruvate kinase M; RNF208: Ring finger protein 208; CI: Confidence interval; Exp: Expression.

### *LTB4R* correlates with prognosis in colorectal cancer patients

To shed light on the clinical implications of the 11 identified prognostic genes, comprehensive expression analyses were conducted on both the GSE164191 and TCGA CRC datasets. Our analyses consistently revealed significant overexpression of these genes in CRC tumor samples compared to normal controls (Figure 4A and 4B). In pursuit of understanding the prognostic significance of these 11 genes, KM survival analyses were executed to assess the impact of their differential expression on OS rates in CRC patients. Notably, among the assessed genes, six emerged with significant prognostic implications (Figure 4C–4H). Specifically, elevated expressions of *INHBB*, *LRRN4*, *METTL27*, *FSTL3*, and *HSF4* were invariably associated with a compromised OS rate. Interestingly, the expression of *LTB4R* initially correlated with a poor prognosis at earlier stages. However, as the disease progressed, the OS rates of patients with elevated *LTB4R* expression began to align with those having low *LTB4R* expression, reflecting the subtle role of this gene at different stages of disease progression. Given this intriguing behavior of *LTB4R* and considering its previously reported association with CRC, albeit without clear functional implications, we selected *LTB4R* for subsequent experimental analyses.

**Figure 5.**
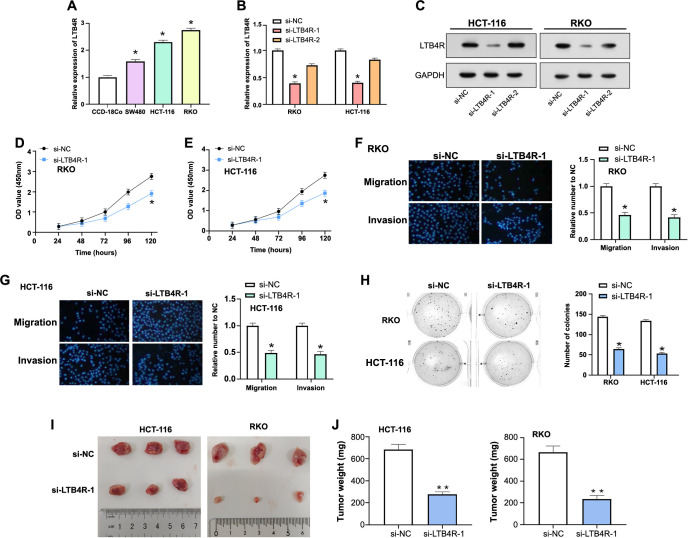
**Functional effects of LTB4R modulation on CRC cell lines and in vivo tumor growth.** (A) qRT-PCR analysis of LTB4R expression levels in CCD-18Co and CRC cell lines, highlighting significant upregulation in CRC cells, especially in HCT-116 and RKO cells; (B and C) qRT-PCR (B) and WB (C) analyses evaluating the LTB4R expression and protein levels in HCT-116 and RKO cells after targeted knockdown using siRNA molecules; (D and E) CCK-8 assay results illustrating a significant decrease in cell viability in RKO (D) and HCT-116 (E) cells following LTB4R knockdown; (F and G) Transwell assay assessing the migration and invasion ability of RKO (F) and HCT-116 (G) cells after LTB4R knockdown, demonstrating reduced migration and invasion abilities; (H) Colony formation assay indicating a reduction in colony-forming ability after LTB4R knockdown; (I and J) In vivo study depicting tumor growth curves and final tumor weights in mice injected with LTB4R knockdown cells, compared to controls. Tumor volume over time (I) and the *y*-axis (J) representing the final tumor weight. *P < 0.05; **P < 0.01. LTB4R: Leukotriene B4 receptor; CRC: Colorectal cancer; qRT-PCR: Quantitative real-time polymerase chain reaction; CCD-18Co: Colon colectomy 18Co; WB: Western blot; siRNA: Small interfering RNA; CCK-8: Counting Kit-8; NC: Negative control; GAPDH: Glyceraldehyde 3-phosphate dehydrogenase; OD: Optical density.

### Regulation of *LTB4R* expression alters colorectal cancer cell behavior and tumor genesis

We began our exploration by quantifying the *LTB4R* expression in the colon colectomy 18Co (CCD-18Co) cell line and various CRC cell lines using qRT-PCR (Figure 5A). A conspicuous upregulation of *LTB4R* was evident in CRC cell lines relative to the colonic epithelial cells. Notably, HCT-116 and RKO cells demonstrated the most pronounced increase, thereby earmarking them for subsequent experiments. Harnessing siRNA molecules, we initiated targeted knockdown assays to discern the functional repercussions of *LTB4R* modulation. Post-knockdown, both qRT-PCR and WB assays validated the suppressed LTB4R expression at transcript as well as protein tiers in HCT-116 and RKO cells (Figure 5B and 5C). Among the utilized siRNAs, si-LTB4R-1 manifested a more pronounced reduction, marking it as an efficacious silencing tool. With the knockdown achieved, a series of assays were deployed to fathom the functional aftermath. The CCK-8 assay revealed a notable decline in cell viability post-*LTB4R* knockdown (Figure 5D and 5E). Further insights emerged from the Transwell assay, which evidenced a marked reduction in the migratory and invasive capabilities of CRC cells post-*LTB4R* silencing (Figure 5F and 5G). Additionally, a decrease in colony formation was observed, indicating reduced colony-forming potential post-knockdown (Figure 5H). Moreover, in an in vivo setting, mice with *LTB4R* knockdown tumors exhibited noticeably slower tumor growth compared to their control counterparts (Figure 5I and 5J). This was further confirmed by the reduction in tumor weight, emphasizing the primary role of this gene in tumorigenesis. This data collectively illuminates the multifarious influence of *LTB4R* in orchestrating critical cellular events, cementing its position as a pivotal determinant in CRC progression.

### *LTB4R* knockdown induces apoptosis and regulates apoptosis-related pathways in colorectal cancer cells

**Figure 6.**
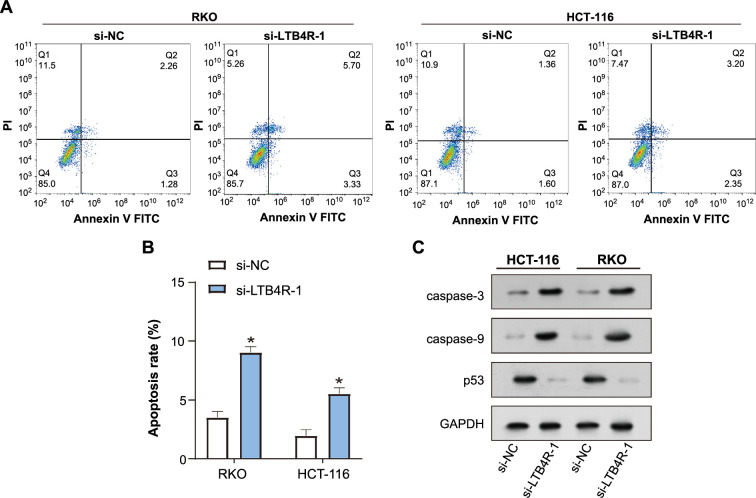
**LTB4R knockdown induces apoptosis in CRC cells and regulates apoptosis-related proteins.** (A and B) Flow cytometry analysis depicting the apoptotic status of RKO and HCT-116 cells after LTB4R knockdown, showing a significant increase in apoptotic events. *P < 0.05. (C) WB analysis evaluating apoptosis-related proteins in HCT-116 and RKO cells post-*LTB4R* knockdown. The representative bands for each group show increased expression of caspase-3 and caspase-9, alongside decreased expression of p53 post-knockdown. LTB4R: Leukotriene B4 receptor; CRC: Colorectal cancer; WB: Western blot; NC: Negative control; PI: Propidium iodide; FITC: Fluorescein isothiocyanate; GAPDH: Glyceraldehyde 3-phosphate dehydrogenase.

The apoptotic status of HCT-116 and RKO cells after *LTB4R* knockdown was assessed using flow cytometry (Figure 6A and 6B). The results showed that *LTB4R* knockdown significantly increased apoptosis, suggesting its potential as a determinant of cell survival. WB analysis was further used to evaluate the expression levels of apoptosis-related proteins after *LTB4R* knockdown (Figure 6C). This revealed a significant increase in caspase-3 and caspase-9 expression, indicating enhanced apoptotic signaling. Conversely, a substantial reduction in p53 expression was observed after *LTB4R* knockdown, suggesting a complex interplay between *LTB4R* and p53-mediated apoptotic pathways.

### *LTB4R* knockdown regulates PI3K signaling pathway and cellular behavior in colorectal cancer cells

**Figure 7.**
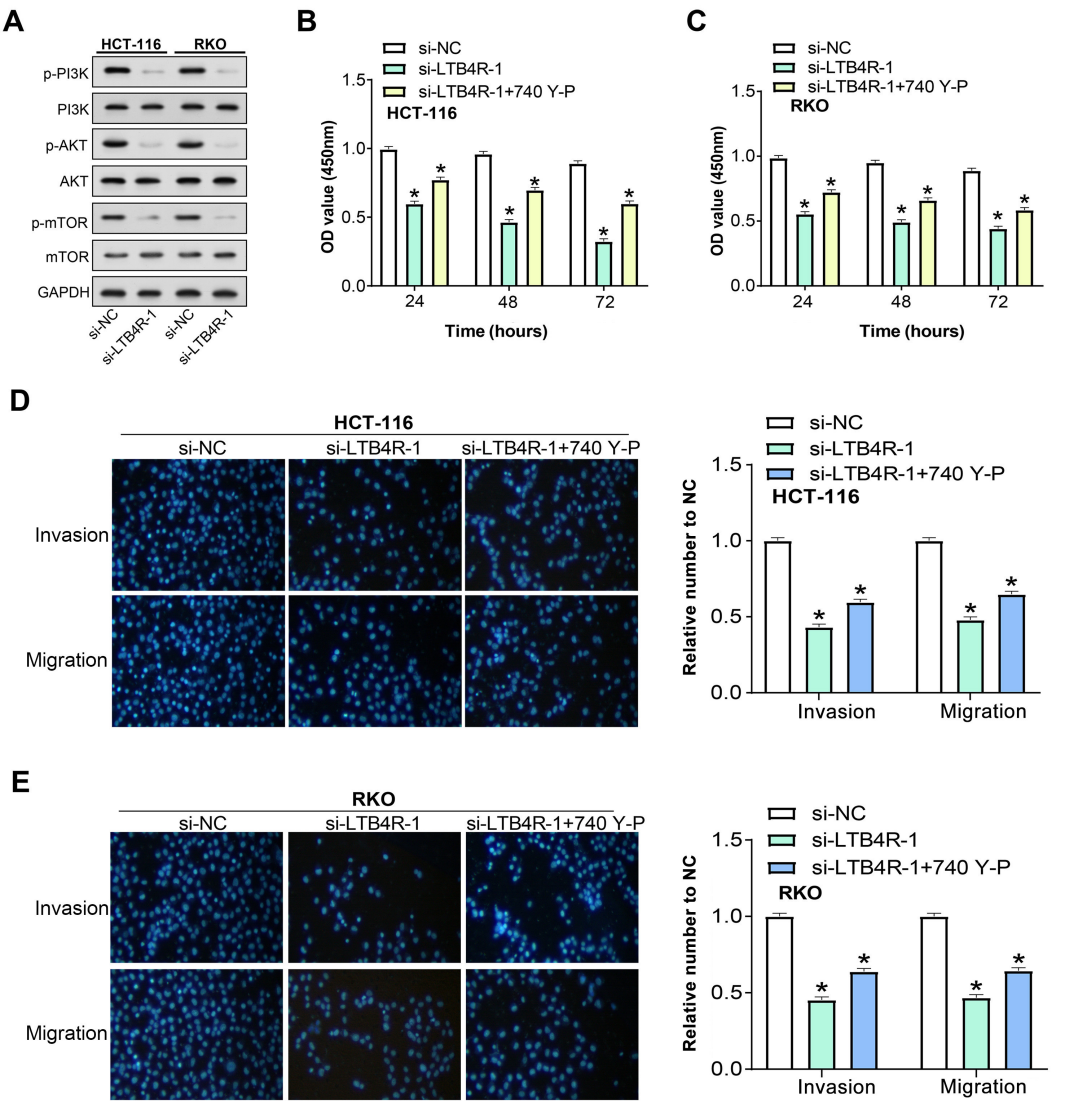
**LTB4R knockdown effects the PI3K signaling pathway and cellular behavior in CRC cells.** (A) WB analysis showing reduced expression of p-PI3K, p-AKT, and p-mTOR in HCT-116 and RKO cells following LTB4R knockdown, indicating the regulatory control of LTB4R on the PI3K/AKT/mTOR axis; (B and C) CCK-8 assay results depicting the effect on cell viability in HCT-116 (B) and RKO (C) cells. PI3K activator (740Y-P) treatment partially recovered cell viability after LTB4R knockdown but did not fully restore it to control levels; (D and E) Transwell assay evaluating invasion and migration capabilities post-LTB4R silencing in HCT-116 (D) and RKO (E) cells. PI3K activator (740Y-P) treatment partially recovered the migratory and invasive abilities but did not fully restore them to control levels. *P < 0.05. LTB4R: Leukotriene B4 receptor; PI3K: Phosphoinositide 3-kinase; CRC: Colorectal cancer; WB: Western blot; p-: Phosphorylated; AKT: Protein kinase B; mTOR: Mammalian target of rapamycin; CCK-8: Counting Kit-8; GAPDH: Glyceraldehyde 3-phosphate dehydrogenase; NC: Negative control; OD: Optical density.

Considering the importance of the PI3K signaling pathway in cellular survival, growth, and proliferation, especially in the context of cancer progression [23], we sought to elucidate the potential interplay between *LTB4R* expression and this critical pathway in CRC cells. WB analysis revealed that silencing *LTB4R* led to a marked downregulation of phosphorylated forms of PI3K, AKT, and mTOR in CRC cells (Figure 7A). This highlighted the potential impact of *LTB4R* on the PI3K/AKT/mTOR signaling cascade, suggesting its multifaceted involvement in CRC pathogenesis. To further discern this relationship, cells were pretreated with the PI3K activator 740Y-P for 4 h before si-*LTB4R* transfection. The consequent observed changes in cellular behaviors after *LTB4R* silencing, depicted in Figure 7B–7E, revealed a striking decline in cell viability, migration, and invasion. Interestingly, the 740Y-P pretreatment only partially ameliorated the inhibitory impacts of *LTB4R* knockdown. Together, these findings suggest that the PI3K signaling pathway may play a mediating role in the *LTB4R*-driven modulation of CRC cellular dynamics. However, its activation cannot fully counteract the consequences of *LTB4R* knockdown, highlighting the overall impact of *LTB4R* in CRC biology.

### The interplay between *LTB4R* silencing, PI3K activation, apoptosis, and related signaling pathways

**Figure 8.**
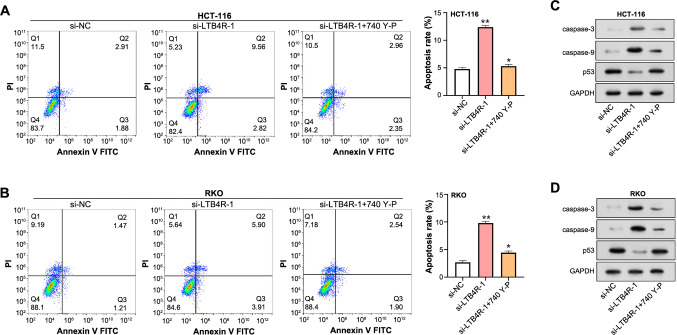
**Effects of LTB4R and PI3K activation on apoptosis and related pathways in CRC cells.** (A and B) Flow cytometry analysis depicting apoptosis levels in HCT-116 (A) and RKO (B) cells after si-*LTB4R* transfection and subsequent 4-hour treatment with a PI3K activator (740Y-P). *P < 0.05. (C and D) WB analysis showing caspase-3, caspase-9, and p53 expressions in HCT-116 (C) and RKO (D) cells following LTB4R knockdown and 740Y-P treatment. LTB4R: Leukotriene B4 receptor; PI3K: Phosphoinositide 3-kinase; CRC: Colorectal cancer; si-RNA: Small interfering RNA; WB: Western blot; NC: Negative control; PI: Propidium iodide; FITC: Fluorescein isothiocyanate; GAPDH: Glyceraldehyde 3-phosphate dehydrogenase.

Following a 4-h pretreatment with 740Y-P, si-*LTB4R* transfected HCT-116 and RKO cells underwent apoptotic assessment using flow cytometry (Figure 8A and 8B). The knockdown of *LTB4R* amplified apoptotic activities, which was only moderately reduced by 740Y-P pretreatment, yet remained above control levels. WB analysis further elucidated the intricate nexus binding *LTB4R* modulation, PI3K pathway stimulation, and apoptotic processes (Figure 8C and 8D). Following *LTB4R* silencing, enhanced expressions of caspase-3 and caspase-9 were documented, accompanied by diminished p53 levels. Although 740Y-P treatment ameliorated the increase in caspase expressions, they were still significantly elevated compared to control levels. Concurrently, p53 levels, albeit elevated post 740Y-P, remained subdued in comparison to baseline values. Collectively, these results delineate the nuanced orchestration of *LTB4R* and PI3K in modulating apoptotic responses in CRC cells.

## Discussion

In the evolving landscape of CRC diagnosis and treatment, LTB4R has emerged as a focal point with transformative potential. Traditional diagnostic methods, including colonoscopy and fecal occult blood testing, have been valuable for early detection but have limitations in providing comprehensive therapeutic guidance [24, 25]. The emergence of molecular targets like *LTB4R*, with its roles in tumorigenesis, inflammation, and metastasis, offers an avenue for more personalized therapeutic strategies. The PI3K/AKT/mTOR signaling pathway exhibits highly conserved properties in eukaryotic cells and it is a key signaling network [26]. This signaling pathway is one of the most commonly dysregulated signals in cancer patients and has a critical impact on tumorigenesis, progression, and response to therapy. This is mainly due to the fact that the PI3K/AKT/mTOR signaling pathway plays an integral role in many cell BPs, including cell growth, metastasis, survival, and metabolism [27]. Our study not only validates the upregulation of *LTB4R* in CRC but also delineates its complex interactions with pivotal signaling pathways, including PI3K/AKT/mTOR and apoptosis-related pathways. Leveraging advanced statistical models such as LASSO Cox regression, we identified a robust 11-gene prognostic signature for CRC, with *LTB4R* as a critical component. These findings could equip clinicians with nuanced prognostic indicators and pave the way for *LTB4R*-targeted therapies, possibly enhancing the efficacy of existing treatments. Importantly, the observed role of *LTB4R* in altering CRC cell behavior substantiates its potential as a multi-faceted therapeutic target. As our understanding of *LTB4R* grows, its ability to revolutionize CRC management, from early diagnosis to personalized treatment, becomes increasingly apparent, underscoring the timeliness and relevance of this research.

In light of our findings, the application of WGCNA in dissecting CRC-related gene modules stands out as a highly informative approach [28, 29]. WGCNA facilitated the construction of a gene co-expression network, allowing us to identify the MEturquoise module, which exhibited a robust positive correlation with CRC pathology. This demonstrates WGCNA’s capability in capturing complex gene interactions and pinpointing the most relevant modules for specific diseases, further extending the landscape of CRC molecular diagnostics and therapeutics. It is noteworthy that our functional enrichment analysis revealed the involvement of pathways like “Glycolysis in senescence” and “Osteopontin signaling.” This is especially compelling given that Amilca-Seba et al. [30] showed that osteopontin acts as a regulator of CRC progression. Furthermore, our study corroborated findings such as the role of genes involved in “Cadherin binding involved in cell-cell adhesion,” echoing the work of Wei et al. [31], who indicated that N-Myc downstream-regulated gene 2 (*NDRG2*) modulates adherens junction integrity to limit colitis and carcinogenesis. Therefore, integrating WGCNA into the analysis of cancer datasets holds promise for elucidating complex gene networks, offering a nuanced understanding that might facilitate the development of targeted and more effective treatment options.

To develop a comprehensive prognostic model, we employed LASSO Cox regression analysis and identified 11 genes with significant prognostic significance in CRC. The risk score model, constructed from these genes, presents compelling predictive accuracy, as evidenced by the AUC values for 1-year, 3-year, and 5-year survival rates. This robust risk model has the potential to guide clinical decision making by providing a more granular understanding of patient survival likelihoods and enabling personalized treatment regimens [32, 33]. The 11 identified genes offer an intriguing mix of well-known and lesser known entities in the context of CRC prognosis. For instance, Yuan et al. [34] previously demonstrated that differential expression of INHBB significantly influences the prognosis of CRC patients and is associated with various oncogenic signaling pathways. Similarly, Xu et al. [35] reported that LRRN4 modulates the malignant phenotype of CRC tumor cells through the rat sarcoma (RAS)/MAPK signaling pathway. Another study highlighted that overexpression of FSTL3 impacts CRC prognosis and can promote epithelial-to-mesenchymal transition (EMT) and aerobic glycolysis via the activated β-catenin pathway, thereby affecting CRC cell invasiveness and metastatic capability [36]. Interestingly, our study also observed that *LTB4R* has a nuanced role in CRC prognosis, becoming less discriminative at later stages. This not only underscores the complexity of the CRC genetic landscape but also stresses the need for multigene models for prognostic predictions.

In building upon the preceding discussions, the multidimensional involvement of *LTB4R* in CRC merits focused attention. Our research establishes *LTB4R* as a critical regulator of not only cellular behaviors such as viability, migration, and invasion but also of pivotal apoptotic pathways. Importantly, the altered expression of caspase-3 and caspase-9 following *LTB4R* knockdown unveils a potential therapeutic avenue for apoptosis modulation. Given that aberrant apoptotic mechanisms are often linked to chemo-resistance [37, 38], targeting *LTB4R* could lead to novel intervention strategies applicable beyond CRC to other malignancies like ovarian and breast cancers. The PI3K/AKT/mTOR signaling pathway is a very important signaling pathway in cancer biology and plays a central role in cell growth, proliferation, metabolism, and survival [39–41]. This pathway is regulated by many growth factors and nutrients and is abnormally activated in many cancer types [42, 43]. Targeting this pathway has shown success in treating various cancers, including CRC. Our finding that *LTB4R* silencing attenuates the activation of this pathway may provide a fresh perspective on CRC therapeutics. Inhibiting *LTB4R* could serve a dual role, pro-apoptotic and anti-proliferative, due to its influence on this critical signaling axis. It is of particular interest that PI3K activation only partially rescues *LTB4R*’s effects, implying that other, as-yet-unidentified signaling molecules may also be at play. Collectively, these findings not only deepen our understanding of CRC pathology but also widen the scope of current research in targeting apoptosis and the PI3K/AKT/mTOR pathway. However, it is worth noting that our study has some limitations. Firstly, it was validated only at the cellular level as we did not carry out the study in an animal model. Furthermore, we did not exhaustively examine the expression of caspase-8 protein, which may lead to some deficiencies in the comprehensiveness and depth of our study. In future studies, we will consider expanding the experimental design to cover animal experiments as well as more comprehensive protein expression analyses to ensure a comprehensive understanding and accurate description of the phenomena under study.

## Conclusion

In conclusion, the research revealed a complex role of *LTB4R* in the pathogenesis of CRC. Through systematic analysis of DEGs, co-expression networks, and functional enrichment, *LTB4R* was identified as a pivotal factor in CRC progression. Our comprehensive studies demonstrated that *LTB4R* exerts multifaceted effects on various cellular behaviors, including cell viability, migration, invasion, and apoptosis. Importantly, the xenograft model provided in vivo validation of the role of *LTB4R* in inhibiting CRC growth. The complex interplay between *LTB4R* and PI3K/AKT/mTOR signaling pathway and apoptosis-related pathways further emphasized its importance in the development of CRC.

## Data Availability

The datasets used and/or analyzed during the current study are available from the corresponding author upon reasonable request.
